# High-resolution long-read sequencing maps genomic Island–associated gene duplication in South African carbapenem non-susceptible *Pseudomonas aeruginosa*

**DOI:** 10.3389/fmicb.2026.1891542

**Published:** 2026-08-05

**Authors:** Rory Cave, Fadheela Patel, Samantha D. Africa, Clinton Moodley, Gert Marais, Widaad Zemanay, Adrian Brink, Hermine V. Mkrtchyan

**Affiliations:** 1 Centre for Innovation in Genomics and Microbiome Sciences, School of Medicine and Biosciences, University of West London, London, United Kingdom; 2 Section of Structural and Synthetic Biology, Department of Infectious Disease, Imperial College London, London, United Kingdom; 3 Division of Medical Microbiology, University of Cape Town, Cape Town, South Africa; 4 Department of Biomedical Sciences, Faculty of Health and Wellness Sciences, Cape Peninsula University of Technology, Bellville, South Africa; 5 National Health Laboratory Service Laboratory, Groote Schuur Hospital, Cape Town, South Africa; 6 Crick Africa Network, Francis Crick Institute, London, United Kingdom; 7 Centre for Infectious Diseases Research in Africa, University of Cape Town, Cape Town, South Africa; 8 Institute of Infectious Disease and Molecular Medicine, University of Cape Town, Cape Town, South Africa

**Keywords:** carbapenem resistant *Pseudomonas aeruginosa*, CRPA, extensively drug-resistant (XDR), long-read whole genome sequencing, South Africa

## Abstract

**Introduction:**

Carbapenem non-susceptible *Pseudomonas aeruginosa* is a major global health threat driven by the spread of high-risk lineages, but the genomic architecture of resistance evolution is often unresolved by short-read sequencing.

**Methods:**

Here, we used long-read whole-genome sequencing to characterize 20 clinical *P. aeruginosa* isolates collected sporadically during routine clinical care as part of the ERACE-PA surveillance programme from a single tertiary hospital in Cape Town, South Africa, between December 2015 and March 2016.

**Results:**

Of these isolates, 18 (90%) were resistant or intermediate resistant to imipenem and/or meropenem. Resistance determinants, mobile genetic elements, and virulence factors were strongly lineage structured. In the high-risk ST233 lineage, resistance was marked by extensive duplication of antimicrobial resistance genes across non-contiguous chromosomal loci. Genes including *dfrB5*, *floR2*, *tet(G)*, *arr-5*, and *sul1* occurred in up to three copies (*sul1*) or two copies (*dfrB5*, *floR2*, *tet(G)*, *arr-5*) across all seven ST233 isolates, distributed on distinct genomic islands, revealing a dispersed amplification architecture that is invisible to short-read approaches because repetitive IS elements and integron boundaries flanking each copy preclude unambiguous mapping and phase-resolved assembly. These loci were linked by shared insertion sequences, class 1 integrons, and transposon-associated segments, consistent with modular transposition and co-integration driving intrachromosomal spread. In contrast, ST273 showed a more plasmid-associated resistance strategy, whereas ST235 displayed more limited duplication despite carrying related mobile resistance modules. Virulence repertoires also differed by lineage: ST233 and ST260 were predominantly ExoS positive, whereas ST273 was ExoU positive.

**Discussion:**

Together, these findings show that carbapenem non-susceptible in South African *P. aeruginosa* is shaped not only by gene acquisition but also by genomic structural redistribution and duplication across separate chromosomal sites, highlighting the value of long-read genomics for resolving genomic structural resistance mechanisms in *P. aeruginosa* surveillance.

## 1 Introduction

*Pseudomonas aeruginosa* (*P. aeruginosa*) is a major public health threat and is a leading cause of hospital and community-acquired infections. This opportunistic pathogen is associated with a devastating range of diseases, including pneumonia and bloodstream infections, predominantly affecting immunocompromised or hospitalized patients (Wilson and Pandey, 2025). The resultant infections are frequently classified as multidrug-resistant (MDR; resistant to ≥2 antibiotic classes) or extensive drug-resistant (XDR resistant to all but ≤2 classes) (Magiorakos et al., 2012). Recognizing the escalating threat, the World Health Organization (WHO) continues to designate carbapenem non-susceptible *P. aeruginosa* as a “high priority” pathogen in its updated 2024 Bacterial Priority Pathogens List, underscoring the urgent need for new therapeutics and enhanced surveillance (WHO, 2024). The clinical challenge is further compounded by the rise of carbapenem non-susceptible, first-line empiric agents in critically ill patients with suspected *P. aeruginosa* infections, driven by loss of OprD, efflux pump overexpression, and hyperproduction of chromosomal *Pseudomonas*-derived cephalosporinase (PDC) enzymes, while the emergence of metallo-β-lactamases (MBLs) presents an additional growing concern (Doi, 2019).

South Africa faces an acute burden of *P. aeruginosa* infections and antimicrobial resistance. In Cape Town, South Africa, *P. aeruginosa* bloodstream infections in children occurred at a rate of 5.4 episodes per 10,000 hospital admissions between 2009 and 2017, with a mortality rate of 24.2% (Dame et al., 2020). Between December 2016 and September 2017, a community-acquired outbreak (3,321 cases) was linked to clonal expansion of sequence type (ST) 303. This lineage harbored genes promoting virulence and survival under environmental stress, including drought conditions (Opperman et al., 2022). In addition, high-risk international clones such as ST111, ST233 and ST235 have also been identified in South Africa, contributing to the spread of MDR and XDR *P. aeruginosa* in healthcare settings; these lineages are frequently associated with resistance to carbapenems and fluoroquinolones, thereby complicating clinical management and infection control efforts (Mudau et al., 2013; Aguilar-Rodea et al., 2017; Treepong et al., 2018; Jung et al., 2024; Flores-Vega et al., 2025).

Prior surveillance at a Cape Town hospital reported high resistance rates to piperacillin (64%), ceftazidime (51%), and imipenem (47%) among clinical *P. aeruginosa* isolates, with 36.8% classified as MDR (Hosu et al., 2021). Overall, 36.8% of isolates were classified as multidrug resistant. The high level of resistance is largely attributable to horizontal transfer of genes, such as acquisition of Extended-Spectrum β-Lactamases (ESBLs) and MBLs. These genes are frequently disseminated via mobile genetic elements such as bacteriophages, genomic islands, integrons, plasmids and insertion sequences, facilitating rapid inter- and intra-species gene exchange (Founou et al., 2018).

Despite the recognized and growing threat posed by *P. aeruginosa*, standard surveillance approaches remain inadequate to fully resolve its extensive genomic complexity. Short-read sequencing platforms frequently fail to capture genomic structural variations, gene duplication, and amplification events, largely due to challenges posed by repetitive flanking regions (Watson et al., 2022). To overcome these limitations and provide a more complete understanding of resistance mechanisms, we applied Oxford Nanopore long-read sequencing to characterize carbapenem non-susceptible *P. aeruginosa* (CRPA) isolates. This integrated genomic approach enabled high-resolution correlation of phenotypic resistance profiles with underlying genetic determinants. Crucially, it revealed for the first time in South African CRPA that resistance gene duplication occurs at non-contiguous chromosomal loci on distinct genomic islands, rather than via simple tandem amplification, a genomic structural complexity that short-read platforms cannot resolve. This distinction has direct implications for understanding resistance evolution and designing surveillance strategies in settings with limited genomic capacity.

## 2 Materials and methods

### 2.1 Sample collection

*Pseudomonas aeruginosa* isolates were collected during the Enhancing Rational Antimicrobials for carbapenem non-susceptible *P. aeruginosa* (ERACE-PA) Global Study (Gill et al., 2022, 2023). A total of 65 carbapenem non-susceptible isolates were collected between December 2015 and March 2016 from patients at Groote Schuur Hospital (GSH), in Cape Town, Western Cape, South Africa. Hospitalized participants with *P. aeruginosa* cultured from any clinical site during routine clinical care were eligible for inclusion in the study. A total of 20 isolates were selected from this collection housed at the National Health Laboratory Service (NHLS), Microbiology, GSH, and were submitted to the Elizayo Biome Sequencing Unit, Department of Medical Microbiology, University of Cape Town, for sequencing and analysis. Of the 20 isolates, one contemporary carbapenem-susceptible and one contemporary carbapenem- intermediate resistant isolate were included to provide context on the resistance profiles circulating at the time of collection. Ethics approval for the study was obtained from the University of Cape Town Human Research Ethics Committee (HREC 413/2024).

### 2.2 Antimicrobial sensitivity testing

Antimicrobial susceptibility testing was performed using the VITEK^®^ 2 automated system (bioMérieux, France) following the manufacturer’s instructions. The system reports categorical interpretations (Susceptible/Intermediate/Resistant) per CLSI/EUCAST breakpoints (Clinical and Laboratory Standards Institute, 2020; European Committee on Antimicrobial Susceptibility Testing, 2020).

### 2.3 Sequencing

The selected twenty *P. aeruginosa* isolates were sub-cultured on blood agar (BA) and DNA extracted using the Quick-DNA Fungal/Bacterial Miniprep Kit (Zymo Research Corp., Irvine, CA, USA) (D6005) according to the manufacturer’s instructions. DNA concentration and purity was assessed using the Nanodrop spectrophotometer (Thermo Fisher Scientific, USA). All DNA samples exhibited acceptable purity ratios (A260/A280: 1.90–1.95). We prepared libraries using the Oxford Nanopore Technologies (ONT) Native Barcoding Kit 24 V14 (SQK-NBD114.24), according to the manufacturer’s ligation-based and native barcoding protocols. Approximately 400 ng of input DNA (10 ng/μL) per sample was used. The sequencing was performed using the R10.4.1 flow cell on the ONT MinION platform.

### 2.4 Genome assembly annotation

Unless otherwise specified, all computational tools were executed using their default parameters. Long-read sequence quality was assessed and trimmed using fastplong (v0.2.2) to remove adapters and low-quality bases below Q15. Genome assemblies were initially generated with Autocycler (v0.3.0) (Wick et al., 2025), and if consensus long-read assemblies were not obtained, Unicycler (v0.5.1) (Wick et al., 2017) was used as a secondary assembler. The genome size and GC content were determined using QUAST v5.3.0 (Gurevich et al., 2013), while completeness and contamination were estimated using CheckM2 (Chklovski et al., 2023). Assemblies (Supplementary Table 1) were included in subsequent analyses if they exhibited a GC content between 65% and 67%, a genome size between 6.0 and 7.8 Mb, completeness greater than 98%, and contamination below 5% (Supplementary Table 2). Genomes were annotated using Bakta (v1.11.4) (Schwengers et al., 2021), and ST were assigned using MLST (v2.19.0)^1^ following the *Pseudomonas aeruginosa* scheme^2^. Genomes were visualized using Proksee (Grant et al., 2023).

Antimicrobial resistance (AMR) genotypes were predicted using AMRFinderPlus (Feldgarden et al., 2021), and virulence-associated genes were identified with Abricate (v1.0.1) using the VFDB database (Liu et al., 2022). AMR gene copy number was confirmed using AMR-STRUCT (v0.1.0). Integrative conjugative was detected using the ConjScan model (Cury et al., 2020) from MacSyFinder (v 2.1.3) (Néron et al., 2023). Plasmid sequences were identified and characterized with MOB-suite (v3.1.9) (Robertson and Nash, 2018), while transposable was annotated using mobileOG-db (Brown et al., 2022). Pairwise nucleotide identity of AMR-containing regions across isolates and between dispersed loci within the same genome was assessed using BLASTn with regions sharing ≥95% identity over ≥95% coverage considered genomically structurally conserved (McGinnis and Madden, 2004). Identification of Gene clustering and visualization between loci were performed with clinker (v0.0.31) (Gilchrist and Chooi, 2021). Phylogenetic trees were constructed using PopPIPE (v1.1.0) (McHugh et al., 2025), and pairwise SNP distances were calculated with snp-dists (0.7.0)^3^.

## 3 Results

### 3.1 Clinical isolate collection and antimicrobial resistance profiles

A total of 20 *P. aeruginosa* isolates were included in this study following quality checks after long-read sequencing. These isolates were carbapenem non-susceptible (resistant or intermediate resistant to imipenem and/or meropenem) (Table 1). The majority were classified as multidrug-resistant (MDR; *n* = 14/20), the remaining 6 exhibited extensively drug-resistant (XDR) profiles (Table 1), highlighting the severe resistance burden in this region. Patients ranged from 7 months to 77 years of age, with isolates more frequently recovered from males (*n* = 14) than females (*n* = 6).

**TABLE 1:** Sample collection and antimicrobial resistance profiles of *P. aeruginosa* isolates.

Sequence name	MLST	Age	Sex	Date collected	Specimen type	AMR category	CIP	CAZ	FEP	AMK	TZP	IPM	MEM	COL
pseudo01	233	33	M	21/02/2016	Superficial swab	XDR	R	R	R	R	R	R	R	S
pseudo02	235	56	F	06/03/2016	Urine	MDR	R	S	S	S	R	S	I	S
pseudo03	260	66	F	02/03/2016	Fluid/Aspirate	MDR	R	S	R	S	I	R	R	S
pseudo04	260	13	F	29/02/2016	Tracheal aspirate	MDR	R	S	I	R	R	R	R	S
pseudo05	233	23	M	08/03/2016	Superficial swab	XDR	R	R	R	R	R	R	R	S
pseudo06	443	38	M	01/02/2016	Tracheal aspirate	MDR	S	R	R	R	R	R	I	S
pseudo07	235	66	M	05/02/2016	Blood culture	MDR	R	S	S	R	I	S	S	S
pseudo08	260	13	F	01/02/2016	Tracheal aspirate	MDR	R	S	I	R	R	R	R	S
pseudo09	233	64	M	05/02/2016	Tissue	XDR	R	I	R	R	R	R	R	S
pseudo10	260	0.68	F	30/01/2016	Sputum	MDR	R	S	I	S	I	R	I	S
pseudo11	233	64	M	12/02/2016	Tissue	XDR	R	R	R	R	R	R	R	S
pseudo12	273	65	M	06/03/2016	Urine	MDR	R	S	S	R	R	R	I	S
pseudo13	260	13	F	14/12/2015	Urine	MDR	R	S	R	S	I	R	R	S
pseudo14	233	26	M	31/12/2015	Fluid/aspirate	XDR	R	R	R	R	R	R	R	S
pseudo15	260	76	M	03/01/2016	Sputum	MDR	R	I	R	S	I	R	R	S
pseudo16	233	77	M	28/01/2016	Blood culture	MDR	R	S	R	S	R	R	R	S
pseudo17	233	63	M	29/01/2016	Tissue	XDR	R	R	R	R	R	R	R	S
pseudo18	260	17	M	28/01/2016	Wound swab	MDR	R	S	R	R	R	R	I	S
pseudo19	260	74	M	01/02/2016	Urine	MDR	R	S	R	R	R	R	I	S
pseudo20	273	46	M	28/01/2016	Superficial swab	MDR	R	I	I	R	R	R	I	S

Antibiotic resistance categories: MDR, multidrug-resistant; XDR, extensively drug-resistant. Antibiotic codes: CIP, ciprofloxacin; CAZ, ceftazidime; FEP, cefepime; GEN, gentamicin; AMK, amikacin; TZP, piperacillin–tazobactam; IPM, imipenem; MEM, meropenem; COL, colistin; ND, non-determined. Antimicrobial Susceptibility was determined by VITEK 2. Red tile, resistant; Yellow tile, intermediate; Blue tile, sensitive.

The isolates belonged to five STs with ST260 (*n* = 8) predominating, followed by ST233 (*n* = 7), ST235 (*n* = 2), ST273 (*n* = 2), and ST443 (*n* = 1). Notably, all but one ST233 isolate displayed an XDR phenotype, exhibiting resistance to nearly all tested antibiotics, yet remained uniformly susceptible to colistin (0/7 resistant). Resistance to ceftazidime (5/7) and amikacin (6/7) among ST233 isolates was variable, underscoring the heterogeneity even within high-risk clones.

Among the MDR isolates (14/20), all remained susceptible to colistin. Variable susceptibility patterns were observed for carbapenems (imipenem: 12/14 resistant; meropenem: 7/14 resistant, 7/14 intermediate), ceftazidime (7/14 resistant), amikacin (8/14 resistant), and piperacillin-tazobactam (9/14 resistant).

### 3.2 Lineage-specific genomic determinants of resistance, mobility, and virulence

**FIGURE 1:**
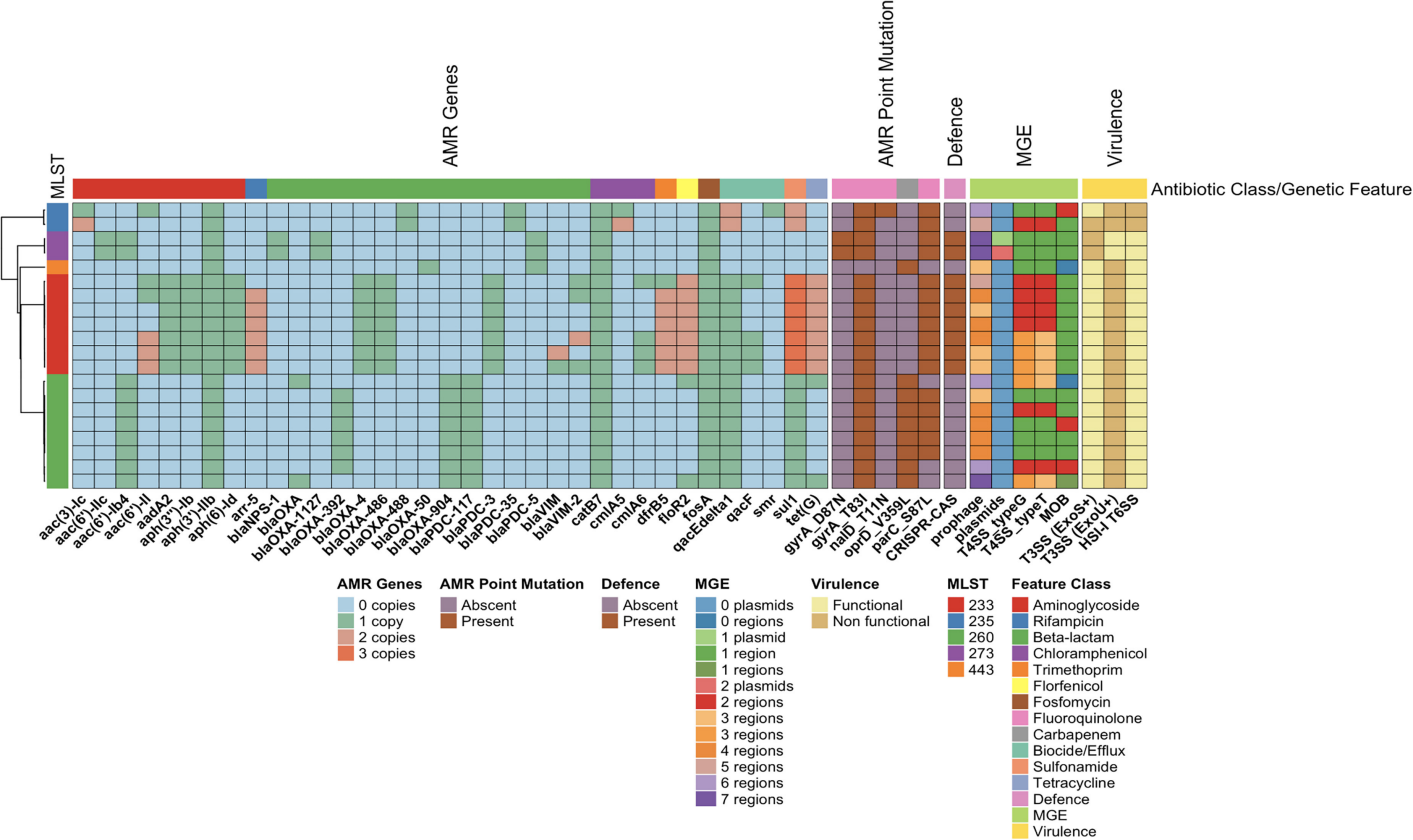
Phylogenetic tree and integrated heatmap summarizing genomic features of all 20 *P. aeruginosa* isolates. Isolates are color-coded by MLST sequence type (ST233 red, ST235 purple, ST260 teal, ST273 gold, ST443 green). The heatmap shows: (i) AMR gene copy number per isolate (0–3 copies, blue–orange scale; (ii) AMR point mutations (present/absent); (iii) CRISPR-Cas system status (functional/non-functional) from MacSyFinder v2.1.3; (iv) mobile genetic element (MGE) count from mobileOG-db; (v) T3SS effector virulence profile (ExoS/ExoU/negative) from Abricate v1.0.1 using the VFDB database. Antibiotic class/feature annotations are shown in the color key at right.

Using twenty whole-genome sequences, we reconstructed a core maximum-likelihood phylogeny (Figure 1) and characterized each isolate for AMR determinants, CRISPR–Cas loci, mobile elements, and virulence factors. Carbapenem-resistance genotypes were lineage-specific: ST233 isolates spanned a broad SNP range (32–136 core SNPs), consistent with heterogeneous acquisition histories. Despite this genetic heterogeneity, all ST233 isolates shared a conserved resistance core (*bla*_*OXA–4*_, *bla*_*OXA–486*_, *bla*_*PDC–3*_), suggesting either selective constraint or location within a highly stable genomic context. Moreover, 2/7 ST233 isolates harbored Metallo-β-lactamases *bla*_*VIM–1*_ and 4/7 harbored *bla*_*VIM–2*_, with *bla*_*VIM–2*_ occasionally duplicated. ST260 showed low pairwise diversity among five isolates (9–16 core SNP differences; *n* = 5/8), whereas three isolates exhibited substantially greater genetic diversity compared with the other five, differing by 87–699 core SNPs. These isolates carried *bla*_*OXA*_, *bla*_*OXA–*392_ (∼75%), *bla*_*OXA–904*_, *bla*_*PDC–*117_, and *opr*D V359L. ST235 was defined by *bla*_*OXA–488*_ and *bla*_*PDC–35*_, ST273 by *bla*_*OXA–*1127_, and ST443 by *bla*_*OXA–*50_ with *oprD* V359L. Non-carbapenem AMR genotypes were similarly lineage-associated.

ST233 also carried multiple aminoglycoside genes (*aac(6′)-Il, aadA, aph(3″)-Ib, aph(3′)-IIb, aph(6)-Id*) and quinolone substitutions (*gyrA T83I*, *parC* S87L), with extensive gene duplication including *dfrB5*, *floR2*, *tet(G), arr-5*, and *sul1*. ST235 showed more limited duplication (*aac(3)-Ic, cmlA5, qacEdelta1, sul1*) and carried *aph(3′)-IIb, fosA*, and quinolone-resistance substitutions, with one isolate also harboring *aac(6′)-Il* and *smr* (Supplementary Table 3). ST260 consistently carried *aac(6′)-Ib4*, *aph(3′)-IIb, catB7, fosA, gyrA T83I, oprD V359L, qacEΔ1*, and *sul1*, with 25% also carrying *tet(G)* and *floR2*. ST273 carried *aac(6′)-IIc, aac(6′)-Ib4, aph(3′)-IIb, catB7, fosA, gyrA D87N, gyrA T83I*, and *parC* S87L, while ST443 carried *aph(3′)-IIb, fosA*, and *oprD V359L*.

CRISPR–Cas loci were detected only in ST233 and ST273. ST233 and ST260 showed no confidently detectable plasmid replicons but harbored multiple prophages; however, plasmid detection may be incomplete because it is assembly- and database-dependent. In contrast, ST273 carried one to two plasmids, including *bla*_*NPS*−1_, supporting potential plasmid-mediated dissemination of β-lactamase genes.

Virulence repertoires were likewise lineage structured. All isolates encoded core functions that underpin persistence and colonization, biofilm formation, adhesion, motility and iron-acquisition systems, but effector profiles of the type III secretion system (T3SS) diverged: T233, ST260, and ST443 were uniformly ExoS-positive. ExoS positivity has been reported to be associated with chronic infection contexts in multiple published studies (Gendrin et al., 2012; Sawa et al., 2014; Nolasco-Romero et al., 2024), although it is not exclusive to chronic infections and can occur in acute presentations (Gendrin et al., 2012). By contrast, ST273 was ExoU-positive, a genotype associated with higher cytotoxic potential in acute severe infections. ST235 isolates were largely ExoS-negative and, importantly, both ST235 genomes in this collection lacked a functional HSI-I type VI secretion system, a deficit that may reduce competitive interbacterial fitness and influence niche occupation (Hood et al., 2010).

### 3.3 Transposon and integron dynamics driving AMR gene duplication

**FIGURE 2:**
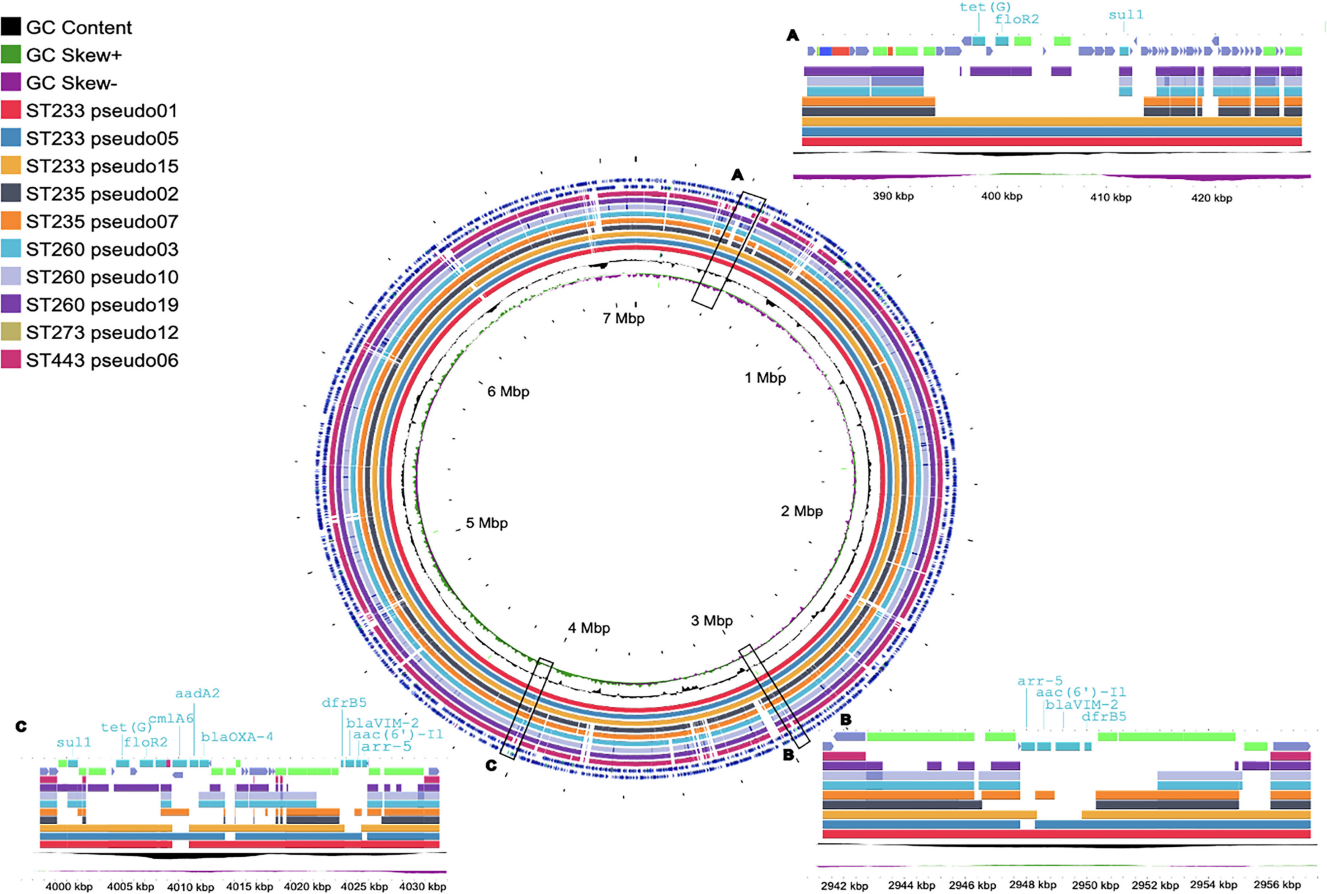
Circular genome map of a representative ST233 isolate (pseudo01) generated with Proksee, showing the three AMR gene duplication loci (A, B, C). The outermost rings show pairwise BLAST alignments of each ST233 isolate to the reference sequence; inner rings show GC content and GC skew. The three inset panels show zoomed linear views of regions A, B, and C, with individual AMR genes and mobile genetic elements labeled. Genomic coordinates and sizes of regions A, B, and C are provided in Supplementary Table 4.

In the ST233 lineage (Figure 2), AMR genes were distributed across three distinct genomic islands. Gene copy numbers were: *sul1* up to 3 copies, *dfrB5* up to 2 copies, *floR2* up to 2 copies, *tet(G)* up to 2 copies, and *arr-5* up to 2 copies. Read-depth profiles at each annotated locus were consistent with single-copy coverage (i.e., not inflated relative to flanking single-copy genes), confirming that duplication reflects bona fide chromosomal dispersion rather than assembly artifact. Duplications occurred at separate loci, apart from *sul1*, where two of the three copies were located within the same locus rather than the same mobile genetic element.

**FIGURE 3:**
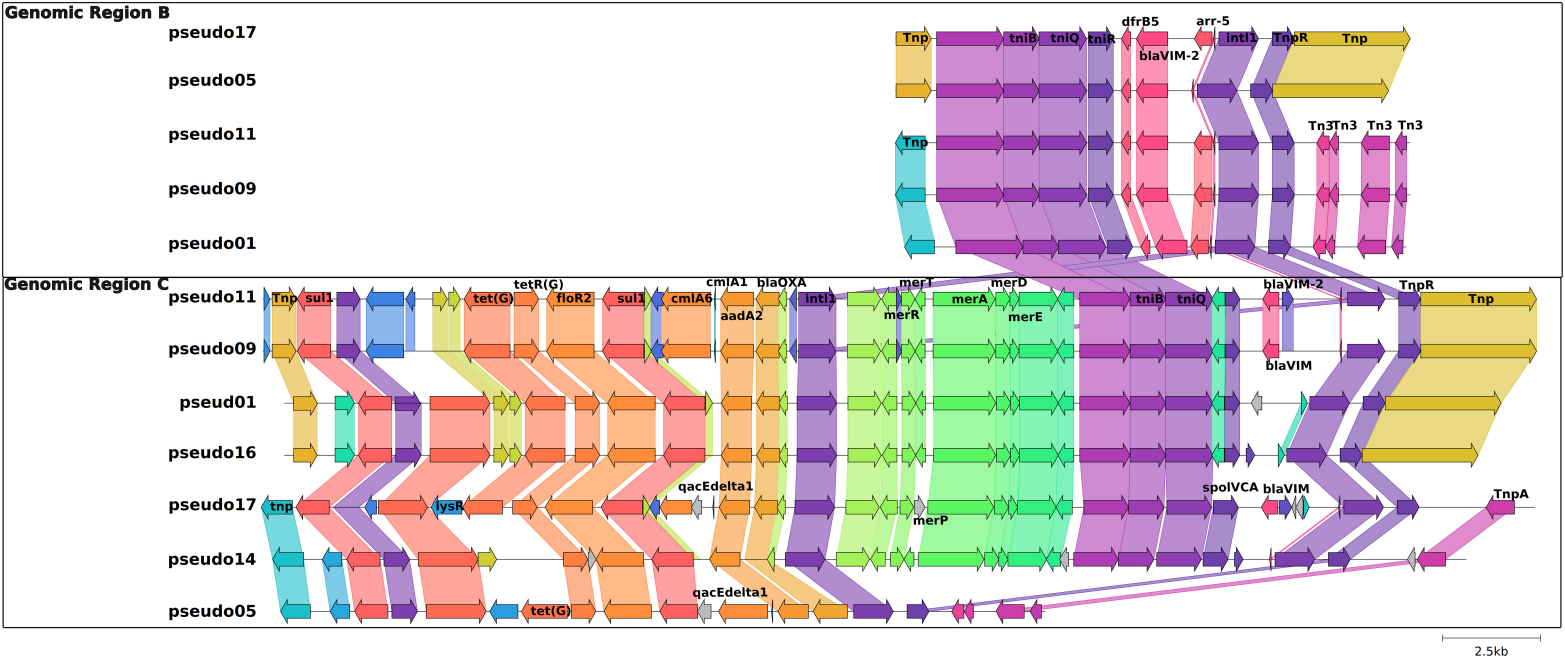
Comparative gene-cluster visualization (clinker v0.0.31) of genomic regions B and C across seven ST233 isolates. Arrows represent annotated genes; arrow color indicates homology group (same color = ≥95% amino acid identity between compared sequences); connecting ribbons are colored by identity score (scale 0–1.0 shown). The *tnsA–tnsB–tnsC–tniQ* cluster (Tn7-like elements), class 1 integron (*intI1*), and Tn3 transposon are highlighted. The scale bar represents 2.5 kb.

**FIGURE 4:**
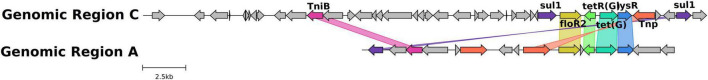
Comparative gene-cluster visualization (clinker v0.0.31) of genomic regions C and A in ST233 isolates showing the shared IS26-like (IS6 family) element and the duplicated *tet(G)*–*floR2*–*sul1* cluster. Ribbon colors and identity scores as in Figure 3. Scale bar 2.5 kb.

In region A, the AMR gene was integrated by transposons between two integrative conjugative elements (ICEs), whereas in regions B and C, AMR genes were independently inserted at distinct loci via transposons. The *tniA–tniB–tniQ–tniR* cluster (Tn7-like elements), a class 1 integron, and a *Tn3* transposon identified in region B were also present in region C, positioned adjacent to *dfrB5*–*bla*_*VIM*_/*bla*_*VIM–*2_–*arr-5* (Figure 3). Furthermore, *tet(G)–floR2*–*sul1* resistance gene cluster was present in both Region A and Region C, consistent with duplication of this element across the two genomic contexts. In Region C, an IS6 family (IS6100) transposase was detected in close proximity to a duplicated *sul1* gene, raising the possibility that IS6-mediated co-integration contributed to the dissemination of this resistance cluster (Figure 4). Region A, by contrast, harbored IS91 family transposases, suggesting that distinct mobile genetic element families may have been involved in shaping each region independently.

**FIGURE 5:**
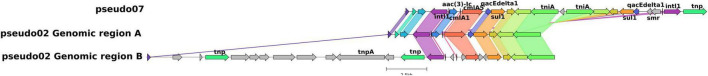
Comparative gene-cluster visualization (clinker v0.0.31) of AMR gene duplication loci in ST235 isolates pseudo07 (genomic region A only; both *sul1* copies co-located within a single genomic island) and pseudo02 (genomic regions A and B; the second *sul1* copy is positioned at a distinct chromosomal locus together with a class 1 integron and Mu transposase element). Ribbon colors and identity scores as in Figure 3. Scale bar 2.5 kb.

By contrast, ST235 isolates exhibited a different pattern. In isolate pseudo07, both copies of *sul1* were located within a single genomic island, whereas in pseudo02 the duplicate copy was positioned in a distinct locus together with a class 1 integron and a *Mu* transposase element (Figure 5). This organization is most consistent with transposon-mediated integration.

## 4 Discussion

Carbapenem-resistant *Pseudomonas aeruginosa* (CRPA) is well documented in South Africa and is frequently associated with internationally recognized high-risk clones, particularly ST233 and ST235. These lineages are major drivers of the global emergence of XDR phenotypes, posing a serious threat to human health.

While previous short-read sequencing studies have identified the presence of key resistance determinants, they have generally lacked the resolution required to resolve the broader genomic context (Kocsis et al., 2021).

The long-read assemblies in this study reveal genomic architectures most consistent with transposable elements having inserted AMR gene cassettes into distinct genomic islands, resulting in duplicated resistance genes at separate chromosomal loci. We emphasize that this is a cross-sectional, descriptive study; the proposed mechanism of transposon-mediated duplication is an inference from genomic structural co-occurrence patterns and cannot be confirmed without temporal sampling, experimental transposition assays, or long-read tracking of insertion junction dynamics across isolates. These duplicated loci appear to arise predominantly through transposon-mediated insertions and co-integration of Insertion Sequence (IS) elements with integron-bearing transposons. This modular organization facilitates both vertical inheritance and horizontal dissemination of multi-gene resistance cassettes and explains why identical resistance clusters can appear at non-contiguous sites within the same lineage.

In other bacterial systems, gene copy-number increases have been experimentally shown to elevate transcript levels and minimum inhibitory concentrations (Souque et al., 2021; Maddamsetti et al., 2024; Silva and Khare, 2024). Whether the duplicated loci identified here produce elevated transcript levels in our isolates remains untested; confirming this relationship would require RT-qPCR or RNA-seq experiments coupled with MIC titrations against the full AMR genotype panel, which are important next steps for future studies (Tafazoli et al., 2025). ST233, in particular, is frequently linked to metallo-β-lactamase carriage (e.g., *bla*_*VIM–*2_) and often harbors integrons and composite transposons that promote horizontal transfer, highlighting the epidemiological significance of these structures (Perez et al., 2014; Zafer et al., 2015; Fortunato et al., 2023). The duplication of *sul1*, *dfrB5*, and *arr-5* in ST233 illustrates the extent of genomic structural redistribution occurring in this lineage; while these genes confer resistance to antimicrobial agents not routinely used for *P. aeruginosa* therapy, their multi-copy architecture is indicative of a broader pattern of mobile element-driven gene amplification that also affects clinically important determinants such as *bla*_*VIM–2*_ and aminoglycoside resistance genes. These duplicated loci are most structurally consistent with transposon-mediated insertions and co-integration of IS elements with integron-bearing transposons, though temporal or experimental evidence confirming this as the generative mechanism is lacking. This modular organization may facilitate both vertical inheritance and horizontal dissemination of multi-gene resistance cassettes; it provides a plausible genomic structural explanation for the appearance of identical resistance clusters at non-contiguous chromosomal sites within the same lineage but does not demonstrate this experimentally. The correlation between copy number and phenotypic resistance, as observed experimentally in other species, suggests that duplicated loci may raise resistance thresholds and reduce treatment success (Maddamsetti et al., 2024; Silva and Khare, 2024). Future studies incorporating MIC titrations against the full AMR genotype panel would help confirm this relationship.

The dispersed chromosomal architecture identified in ST233 differs from the most prevalent AMR gene amplification mechanisms reported in other Gram-negative pathogens in several key respects. Comparative genomic analyses of ESKAPE pathogens have increasingly highlighted the vast genetic repertoire and extensive metabolic pathways that *P. aeruginosa* leverages to develop robust antimicrobial resistance, distinguishing its adaptive strategies from those of other nosocomial threats (Sahayarayan et al., 2024). Tandem gene amplification mediated by replication slippage or homologous recombination between direct repeats, the predominant mechanism described in *K. pneumoniae* (e.g., tandem *bla*_*KPC*_ arrays) and *Acinetobacter baumannii* produces adjacent copies at a single chromosomal region (Lam and Hamidian, 2024; Silva and Khare, 2024; Ye et al., 2025). The ST233 architecture instead features physically separated loci on distinct genomic islands, implying transpositional reinsertion as the generative mechanism. Furthermore, the co-duplication of multi-gene cassettes (*tet(G)*–*floR2*–*sul1*; *dfrB5*–*bla*_*VIM–2*_–*arr-5*) rather than individual genes, and the embedding of these cassettes within ICE-flanked genomic islands rather than on plasmids, represents a structural configuration that has not, to our knowledge, been previously described in *P. aeruginosa* or in Gram-negative pathogens more broadly at this level of resolution.

We acknowledge that the isolate collection is temporally restricted to 2015–2016. However, the lineages and genomic structural resistance mechanisms characterized here remain clinically relevant: ST233 and ST235 continue to circulate in South Africa and globally as high-risk clones (Jung et al., 2024; Flores-Vega et al., 2025), and recent genomic surveillance across multiple countries confirms that mobile-element-driven AMR gene amplification is an ongoing and expanding phenomenon in CRPA (Karampatakis et al., 2025). Recent genomic surveillance in 2023 from ICU admission screening in Hanoi, Vietnam further highlights global dissemination *P. aeruginosa* high-risk clones carrying metallo-β-lactamases, predominantly *bla*_*NDM–1*_, with widespread resistance to newer anti-pseudomonal agents, including ceftazidime–avibactam and ceftolozane–tazobactam emphasizing the clinical challenge by metallo-β-lactamases lineages (Göpel et al., 2025). This pattern is mirrored in Italy, where XDR ST235 predominated and half of the isolates exhibited next-generation cefiderocol resistance, underscoring continuous global resistance evolution (Pantanella et al., 2026). Future prospective genomic surveillance studies would directly address whether these genomic structural architectures have persisted, evolved, or been replaced in current clinical isolates. Individual patient outcomes, mortality rates, and treatment responses were not available to this study under the current ethics framework, which covers anonymous biorepository isolates without clinical record linkage. This limits the direct clinical translational inference. Future studies integrating whole-genome sequencing with matched clinical outcome data would permit direct interrogation of whether genomic structural resistance features such as AMR gene duplication correlate with treatment failure, length of stay, or mortality, as has been demonstrated for other high-risk clonal lineages. The study included only one carbapenem-susceptible isolate as a comparator, limiting our ability to identify genomic features that are exclusive to the carbapenem non-susceptible phenotype. A comparative genomic design with a well-powered susceptible comparator group from the same hospital and time period would enable formal identification of resistance-associated genomic elements and is recommended as a design priority for future surveillance studies at this institution. Without clinical epidemiological linkage (ward, admission dates, patient identifiers), we cannot distinguish clonal transmission from repeated independent introductions, and future studies coupling genomic surveillance with patient movement data would resolve this question.

## 5 Conclusion

Overall, the co-occurrence of integrons, Tn7-like clusters, and insertion elements in these high-risk STs illustrates how modular mobile elements shape the resistome, enabling rapid phenotypic shifts with tangible clinical consequences. Integrating structural genomics into routine surveillance is therefore essential to anticipate emerging resistance architectures and guide antimicrobial stewardship and infection-control strategies.

## Data Availability

The datasets presented in this study can be found in online repositories. The names of the repository/repositories and accession number(s) can be found below: https://www.ebi.ac.uk/ena, PRJEB113414.

## Footnotes

1
https://github.com/tseemann/mlst

2
https://pubmlst.org

3
https://github.com/tseemann/snp-dists
